# Performance and Exergy Analyses of a Solar Assisted Heat Pump with Seasonal Heat Storage and Grey Water Heat Recovery Unit

**DOI:** 10.3390/e23010047

**Published:** 2020-12-30

**Authors:** Primož Poredoš, Boris Vidrih, Alojz Poredoš

**Affiliations:** 1 Laboratory for Refrigeration and District Energy, Faculty of Mechanical Engineering, University of Ljubljana, 1000 Ljubljana, Slovenia; boris.vidrih@fs.uni-lj.si; 2 Slovenian Energy Association, 1000 Ljubljana, Slovenia; alojz.poredos@sze.si

**Keywords:** exergy efficiency, grey water heat recovery unit, seasonal heat storage, solar-assisted heat pump

## Abstract

The main research objective of this paper was to compare exergy performance of three different heat pump (HP)-based systems and one natural gas (NG)-based system for the production of heating and cooling energy in a single-house dwelling. The study considered systems based on: 1. A NG and auxiliary cooling unit; 2. Solely HP, 3. HP with additional seasonal heat storage (SHS) and a solar thermal collector (STC); 4. HP with SHS, a STC and a grey water (GW) recovery unit. The assessment of exergy efficiencies for each case was based on the transient systems simulation program TRNSYS, which was used for the simulation of energy use for space heating and cooling of the building, sanitary hot water production, and the thermal response of the seasonal heat storage and solar thermal system. The results show that an enormous waste of exergy is observed by the system based on an NG boiler (with annual overall exergy efficiency of 0.11) in comparison to the most efficient systems, based on HP water–water with a seasonal heat storage and solar thermal collector with the efficiency of 0.47. The same system with an added GW unit exhibits lower water temperatures, resulting in the exergy efficiency of 0.43. The other three systems, based on air–, water–, and ground–water HPs, show significantly lower annual source water temperatures (10.9, 11.0, 11.0, respectively) compared to systems with SHS and SHS + GW, with temperatures of 28.8 and 19.3 K, respectively.

## 1. Introduction

The reducing of energy consumption and the lowering of greenhouse gas emissions play an important role in the sustainable development of future society. The measures taken so far to achieve the energy and environmental targets by 2030 and 2050 are showing positive results but are not yet ambitious enough or sufficiently effective.

Energy consumption for heating and cooling in buildings and industry accounts for almost half of total energy consumption in all sectors [1]. Therefore, the heating and cooling sector has a huge potential to reduce primary energy consumption and achieve sustainable development objectives. The share of fossil fuels is almost 75%. The share of renewable energy sources (RES) for heating and cooling can be increased by using solar heat directly for heating or cooling as “free cooling”, but to a limited extent due to geographical and climatic conditions. Potential fluctuations can be compensated by different types of electrical energy storage, e.g., electrochemical energy storage (batteries). By balancing the daily fluctuations of the sources, we can significantly reduce the required installed capacity of heating or cooling systems. Seasonal storage of heating or cooling energy enables us to transfer energy from the time of surplus to the time of deficit. In most cases, a heat pump (HP) can be used very efficiently to fully cover the demand for heating or cooling energy. In this way, all types of heat, including low-temperature heat, can be raised to the temperature level suitable for the heating system. A heat pump with minimal exergy consumption produces heat for space heating or sanitary hot water (SHW). Much of the heat produced by a HP is actually anergy drawn from the environment or other sources of waste or surplus heat. This is particularly evident when low temperature heating systems are used with energy efficient systems for distributing heat, such as underfloor heating in low energy houses. A thorough analysis of the energy efficiency of different heat sources and heating systems and their optimal control was carried out by Kazanci et al. [2] and Razmara et al. [3].

The building envelope can be regarded as the boundary of the system under consideration. In order to minimize the energy consumption in a building, it is important to minimize the energy flows beyond this system boundary. This can be achieved by the optimal use of internal heat sources. Various heat sources in the building are known, such as household appliances, electronic devices and wastewater. Following the definition of Sutherland et al. [4], in the context of this study we have restricted ourselves to grey water, which does not include black water, i.e., sanitary wastewater.

In this paper, possible exergy, and, to some extent, energy savings for heating and cooling a building using RES and minimal energy flows across system boundaries were analyzed. In the literature, we found similar partial studies on this research topic. Dennemand et al. [5] conducted an experimental study on a photovoltaic/thermal (PV/T)-assisted brine-to-water HP for sanitary hot water (SHW) production. The results of the 9-month measurements showed that the non-insulated PV/T module heats the hot water tank to ambient temperature even during the time without direct solar radiation.

The energy efficiency of PV/T-assisted HP for heating a single-family house was carried out by Hengel et al. [6]. Within the scope of their research work, they performed a simulation of a solar-assisted HP heating system in combination with a horizontal ground collector. The main results were the experimentally determined coefficient of performance (COP) and the seasonal performance factor (SPF). Based on the results, a significant positive effect of the heat storage tank on the energy efficiency of the HP was found. A field test with a solar integrated air source heat pump using R407C and with underfloor heating was conducted by Dong et al. [7]. Compared to a single air source heat pump, the former system achieved 7.9% higher exergy efficiency (76.8% vs. 71.1%). A rather low exergy efficiency in the case of a ground source heat pump with wall heating with a value of 27.4% was reported by Akbulut et al. [8]. The main reason for such an efficiency is the relatively low compressor efficiency due to the frequent activation of the compressor depending on the outdoor temperature. In the field of process heat generation, Suleman et al. [9] evaluated an integrated solar heat pump system with an exergy efficiency of 35.7%. Experimental studies of PV/T-assisted HPs in combination with a seasonal heat storage system were conducted by Naranjo-Mendoza et al. [10]. The study shows that the seasonal heat storage system significantly increases the efficiency of the heating system during the entire heating season. However, the need to improve the control to prevent extreme temperatures of the inlet liquid to the evaporator was pointed out. Ezzat et al. [11] performed energy and exergy analyses for a multigeneration heating, cooling, and electrical power generation system powered by renewable energy sources, namely geothermal and solar. They report an overall exergy efficiency of 42.8%.

Several authors—Wong et al. [12], Ni et al. [13], and McNabola et al. [14]—investigated the effects of heat recovery from waste and grey water in buildings on energy use for heating purposes. They found varying proportions, from negligible to significant, of the coverage of heating needs depending on climatic conditions, the type of thermal characteristics of the building and the activities taking place in the building. Sun et al. [15] performed an exergy analysis comparing a multifunctional and a conventional heat pump (air conditioner). The multifunctional HP provided both cooling energy for space cooling and SHW by utilizing condensation heat. Compared to the conventional system, the multifunctional HP reduces the exergy loss ratio by 9.06%.

In the literature, there are some exergy studies of PV/T-assisted HPs [16,17] in combination with heat storage tanks [18], and general exergy analyses of different building heating systems [19,20,21].

In this paper, the results of investigations on thermal properties and exergy analyses of a complex system of a solar assisted HP with seasonal storage and grey water heat recovery system are presented, which have not been found in the available literature.

## 2. Description of Analyzed Systems

### 2.1. Case Building

**Figure 1:**
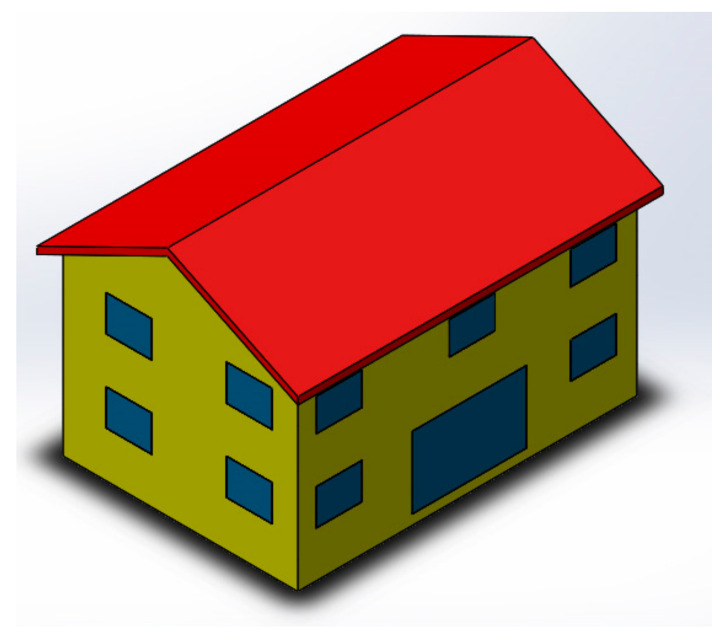
Three-dimensional scheme of a case building.

The case study building is a two-story single-family house (Figure 1) with plan dimensions of 8.2 m × 11.3 m. It has 185 m^2^ of living area. The double-pitched roof has an N–S orientation. Analysis was performed for a low-energy building with specific heating demand 25.8 kWh/m^2^a. The U values of building envelopment are summarized in Table 1.

Four people were considered to occupy the house between 18^00^ and 7^00^ the following day on weekdays, and between 00^00^ and 24^00^ on weekends. Necessary indoor air quality was provided with mechanical ventilation system. Exterior shades with shading factor S_f_ = 0.7 were used on all windows and on the green house when solar radiation on the horizontal plane was larger than 300 W/m^2^ and T_in_ > 24 °C.

**Figure 2:**
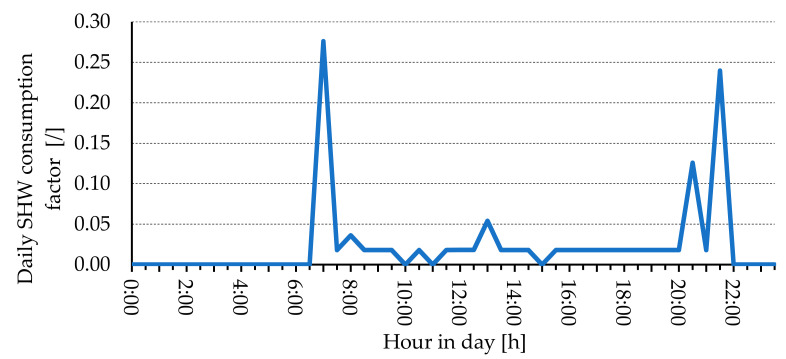
Daily schedule of SHW consumption [22].

The daily sanitary hot water (SHW) used is set to 120 L/day. Figure 2 shows the daily profile of SHW use for the weekday day and the weekend. The temperature of the SHW is set to 55 °C, while the temperature of the sanitary cold water (SCW) is 12 °C. 

### 2.2. Description of Heating and Cooling Energy Production Systems

**Figure 3:**
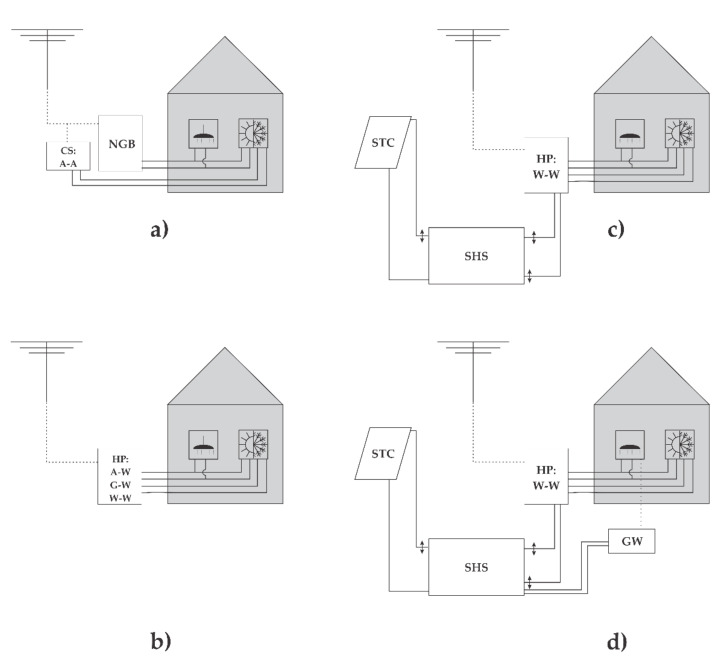
Four different analyzed cases: (**a**) Heating system, based on NG boiler and cooling system, and based on auxiliary air–air vapor compression cooling device; (**b**) heating and cooling system, based on air—water, water—water and ground—water HP; (**c**) heating and cooling system, based on water—water HP, SHS and STC; (**d**) heating and cooling system, based on water—water HP, SHS, STC and GW recovery unit.

The presented study was performed for four different systems for heating and cooling energy production, as shown in Figure 3. The first one, denoted by (a), represents a heating system based on NG. The cooling system, in this case, is based on an auxiliary air–air vapor-compression cooling device. The second, third, and fourth systems, denoted by (b)–(d), represent combined heating and cooling systems that are based on HPs. The most basic version among these systems is the variant (b), with only HP for producing the heating and cooling energy. The third variant, denoted as (c), also encompasses a seasonal heat storage (SHS) unit, combined with a solar thermal collector (STC), while in the case of the fourth variant, denoted as (d), a grey water (GW) recovery unit has also been connected to an SHS.

In the subsequent chapters, all systems will be denoted as:

- GB: Building service systems based on a natural gas boiler for heating purposes and an auxiliary air–air vapor-compression cooling device for cooling purposes (Figure 3a).
- HP A–W, W–W, G–W: Building service systems based on air–water, water–water and ground–water heat pumps for heating and cooling purposes (Figure 3b).
- SHS(STC)–HP W–W: Building service systems based on a water–water heat pump for heating and cooling purposes, seasonal heat storage and solar thermal collectors (Figure 3c).
- SHS(STC + GW)–HP W–W: Building service systems based on a water–water heat pump for heating and cooling purposes, seasonal heat storage, solar thermal collectors and grey water recovery unit (Figure 3d).

The working characteristic of heat and cold generators are presented in Table 2. The performance of the HPs (output thermal power, COP and electric power) were modelled as a function of the heating temperature (i.e., water outlet temperature, which is inlet water temperature to the heating system) and the heat source temperature (i.e., ambient (air) temperature in the case of A–W, ground temperature in the case of G–W and ground water or water from SHS temperature in the case of W–W HP). The performance of the gas heating system (efficiency and electric power) was modelled as a function of the annual efficiency. In the case of NG boiler, the auxiliary vapor-compression cooling device was considered to produce the necessary cooling energy. The electrical power consumption of the water pump in the case of ground–water HP was 50 W and in the case of a water–water HP was 350 W. 

#### 2.2.1. Building Heating and Cooling Energy Distribution Systems

For building space heating, the floor heating system was considered. In each floor, we assumed six pipe loops. The temperature of heating media was controlled as a function of the ambient temperature. The electric power consumption of the circulation pump was 50 W (considered for all four analyzed systems) [10]. For space cooling, fan coils with a standard 7/12 °C regime have been considered. 

#### 2.2.2. Building Ventilation System

The mechanical ventilation system consists of an integrated highly efficient heat recovery unit with efficiency of 0.91. When the building is considered occupied, the nominal air-change rate (ACH) is determined by minimal hygienic requirements according to CR 1752 [25]. In the case of a single-family house, the minimum ACH is 0.5 h^−1^, but during the unoccupied hours the minimum ACH is reduced to 0.2 h^−1^. The mechanical ventilation system also provides more intensive ventilation of the building (ACH up to 1.3 h^−1^), in the case of night of free cooling operation mode. The electric power consumption of the fan is dependent on the volumetric flow. The specific value of 1 kWs/m^3^ is assumed. 

#### 2.2.3. Sanitary Hot Water System

For sanitary water heating, a 300 L water storage with 5 cm insulation is assumed. Due to legionella prevention measures, a one-week overheating of the water storage tank for 1 h by superheating the SHW to 62 °C has also been decided. Heat losses of both the water storage and connecting pipelines were considered. The electric power consumption of the circulation pump was also regarded for all four analyzed systems [22]. 

#### 2.2.4. Solar Thermal Collector System

The solar thermal collector system consists of flat plate collectors located on the south-facing part of the roof with a 45° slope. In the presented case, the total area of solar collectors was 20.6 m^2^ in the case of the SHS (STC)–HP W–W system and 10.4 m^2^ in the case of the SHS (STC + GW)–HP W–W heating system. All other characteristics of solar thermal collectors are shown in Table 3.

#### 2.2.5. Seasonal Heat Storage

To ensure higher efficiency of the system, heat is stored directly in the seasonal heat storage with a capacity of 85 m3 in the case of the SHS (STC)–HP W–W system and 95 m3 in the case of the SHS (STC + GW)–HP W–W system. The storage tank was installed in the ground. The average tank loss coefficient per unit area was 0.1 W/m^2^K. During the SHS operation, we assumed an electric power consumption of the circulation pump of 50 W and a 93% effectiveness of the heat exchanger [26]. 

#### 2.2.6. Grey Water Heat Recovery Unit

To simulate reuse of heat from grey water, a grey water heat recovery unit, based on the spiral heat exchanger, was used, with an effectiveness of 85%. The daily amount of grey water, with a temperature of 42 °C [27], was set to 120 L/day. The daily profile of grey water use is the same as SHW. To ensure higher efficiency of the system, heat is stored directly in the seasonal heat storage. During the grey water system operation, the electric power consumption of the circulation pump of 50 W has been assumed. The operation of the system depends on the temperature of the storage tank and it occurs when the temperature of the heat storage is 3 K lower than the water from the heat storage.

### 2.3. Description of the Simulation Framework

**Figure 4:**
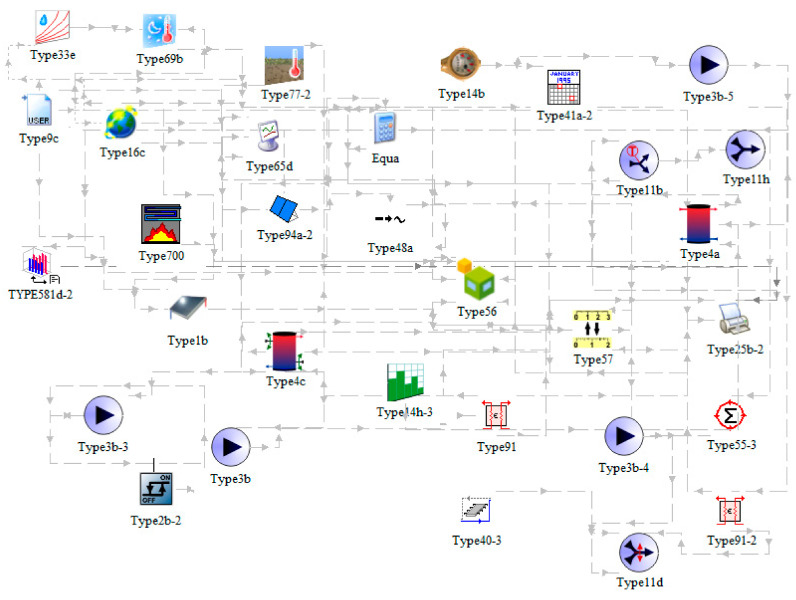
Block scheme of TRNSYS simulation framework.

The transient systems simulation program TRNSYS (TRNSYS, 2000) was used for simulation of energy use for space heating and cooling of the building, SHW production, and the thermal response of the SHS and STC system. TRNSYS is a simulation tool that allows simulations of connected complex systems, as exemplified in this article. Its programming framework is based on a block diagrams, where several systems are connected together, as depicted in the Figure 4.

Analyzed period was 1 year from 1 January to 31 December. The default simulation time step of 1 hour was considered in the simulation. Meteorological data (ambient temperature, horizontal solar radiation, wind speed and relative humidity) were taken from the test reference year (TRY) database for the city of Ljubljana (N 46 3+ 23″, E 14 30′ 29″). All the main components employed in the TRNSYS simulation are listed in Table 4.

## 3. Exergy Efficiency Calculations

Exergy efficiency for a steady-state process occurring in a heating and/or cooling system can be calculated based on the following equation [28]:

(1) $$\eta_{\mathrm{ex}}\;=\;\frac{\mathrm{Exergy}\;\mathrm{in}\;\mathrm{product}\;\mathrm{outputs}}{\mathrm{Exergy}\;\mathrm{in}\;\mathrm{inputs}}$$

Based on this equation, overall exergy efficiency, considering heating and cooling processes, has been calculated as well as partial exergy efficiencies for space heating and cooling and sanitary hot water production. Overall exergy efficiency of all systems has been calculated on a basis of the following equation:

(2) $$\eta_{\mathrm{ex},\mathrm{ov}}\;=\;\frac{\sum E_{h}+\sum E_{shw}+\sum E_{c}}{\sum E_{in}}$$

where $\sum E_{h}$, $\sum E_{shw}$, $\sum E_{c}$ denote sum of exergy in product outputs over a time period, i.e., exergy of heat, required for space heating and sanitary hot water production, and exergy of cooling energy, required for space cooling. The value $\sum E_{in}$ denotes exergy in inputs, i.e., electrical energy in the following cases: 1. HPs for heating (space heating of building and SHW production) and cooling purposes; 2. In the case of pumps connecting SHS with ST collectors and GW; 3. Exergy of the energy carrier in the case of natural gas heating systems. All the partial exergy efficiencies have been calculated in the same manner, excluding parts that are not used during the calculations.

The quantity of exergy of the heat can be evaluated by the work output of a Carnot heat engine, which operates between the hot reservoir and the ambient temperatures:

(3) $$E_{h/\mathrm{shw}}\;=\;\left(\frac{T_{h/\mathrm{shw}}{\;-\;T}_{a}}{T_{h/\mathrm{shw}}}\right)Q_{h/\mathrm{shw}}$$

Exergy of cooling energy has been calculated using a similar equation:

(4) $$E_{c}\;=\;\left(\frac{T_{a}\;-{\;T}_{c}}{T_{c}}\right)Q_{c}$$

In case of the heating system based on NG, the exergy in inputs, i.e., the exergy of heat, produced by a NG boiler, has been calculated by considering the following fact: according to Rant [29], the ratio of difference of specific exergy and superior calorific value in relation to superior calorific for methane (CH_4_) at the reference temperature of 25 °C is:

(5) $$\frac{e_{{\mathrm{CH}}_{4}}\;{-\;H}_{{s,\mathrm{CH}}_{4}}}{H_{{s,\mathrm{CH}}_{4}}}=\;-\;0.0815$$

This ratio shows that the typical exergy efficiency of heat produced by a NG boiler is (1–0.0815), i.e., approximately 92%.

## 4. Results and Discussion

In this section, the results of the performance and exergy analysis for five different cases of heat and cooling energy production with different systems in a four-family house are presented below.

### 4.1. Performance Analysis of Building Simulation

In order to determine the exergy efficiencies of all presented systems, several other parameters had to be determined by using the simulation tool TRNSYS.

**Figure 5:**
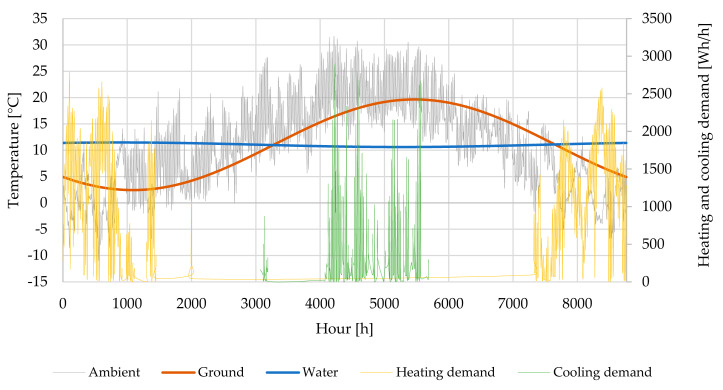
Annual temperature variation of air (ambient), ground (brine) and ground water. Annual heating and cooling demand variation of a building.

Systems B, C and D considered heat pump systems for heating and cooling energy production. The heat source for System B (heat pumps A–W, W–W and G–W) were ambient air, ground water and ground (brine). Its annual temperature variation is shown in the Figure 5. In relation to ambient air temperature, the annual heating and cooling demand is depicted in Figure 5. As specific heating demand was low, based on the overall heat transfer coefficients of envelope and other building elements (Table 1), from April to October there was no heating in order to sustain the inner air temperature within the prescribed range (Table 2). Cooling in the building, on the other hand, took place between May and August.

**Figure 6:**
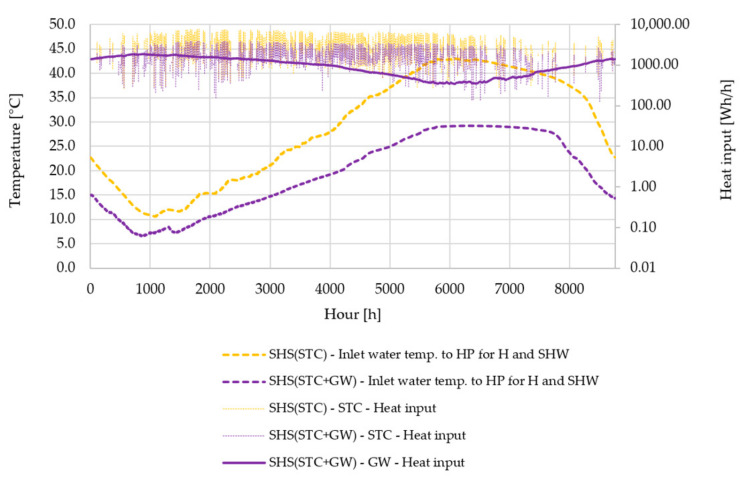
Annual variation of inlet water temperature to HP from SHS for heating and SHW and heat input into SHS in the case of a system with STC and a system with STC and GW. Annual variation of heat input into SHS in the case of a system with STC and a system with STC and GW. The secondary vertical axis (Heat input) has a logarithmic representation for the sake of the diagram comprehensibility.

The heat source for Systems C and D was water inside the SHS. As already explained in Section 2, the heat from solar thermal collectors and the grey water recovery unit was transferred to a SHS, from where this heat was transferred to HP W–W. The inlet water temperatures for System C (SHS + STC) and System D (SHS + STC + GW) are shown in Figure 6. One can identify the difference of inlet water temperature between both systems in favor of System C (only STC, no GW). The main reason for this observation is the lower temperature input when utilizing heat from grey water, and also the lower area of STC in this case. The difference is more pronounced in the region with the higher values of solar radiation (between 1500 and 6000 h), as more heat with higher temperature is fed into the SHS. The annual inlet water temperature variation also shows a discrepancy between the building heating need and the provided heat input from the STC. At the end of autumn (6000 h), the temperature slowly decreases, while in the case of System D, the start of the decreasing point is shifted towards 8000 h due to the GW share of input heat. At around 1000 h, the temperature starts to increase due to both the lower need for building heating and the higher amount of heat input from the STC.

The heat input of both the STC and GW for both Systems C and D can also be observed in Figure 6. Compared to the GW heat input, the STC heat input shows a higher rate of discontinues in providing heat during the months around winter.

**Figure 7:**
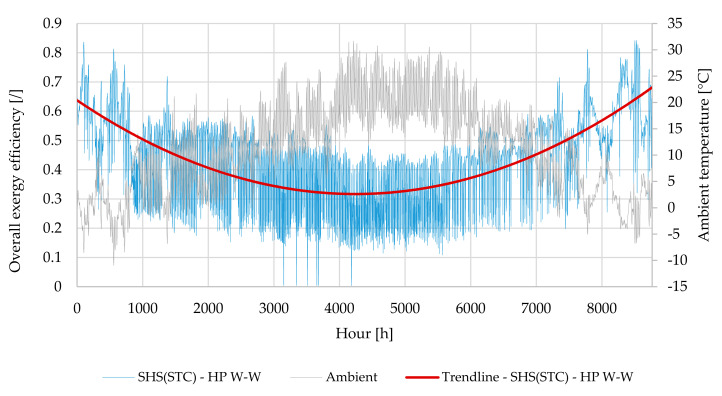
Annual variation of hourly averaged exergy efficiency of a system SHS (STC)–HP W–W.

The overall exergy efficiency and the partial exergy efficiencies for space heating and cooling and SHW production were calculated on the basis of the equations presented in Section 3, either annually or monthly averaged. The calculation of the overall exergy efficiency for the system SHS (STC)–HP W–W for a time step of one hour is shown in Figure 7. The exergy efficiency is also shown with a trend line from which we can see that it has the highest values in the months with the lowest ambient temperature. This is fully in line with the expectations based on the definition of exergy efficiency, since exergy is defined on the basis of the dead state, which in our case is at ambient temperature and pressure.

The greater the temperature difference between the environment and water for heating or cooling purposes, the greater the proportion of exergy is in the end product. On the other hand, it is a fact that the COP of a heat pump is highly dependent on the temperature of the heat source and heat sink. The greater its temperature difference, the smaller the COP of a HP. Due to this fact, in the following chapters we will show not only the overall exergy efficiency but also the partial exergy efficiencies of all systems described: exergy efficiency for space heating, domestic hot water production and space cooling.

### 4.2. Exergy Efficiency—Annualy Averaged

**Figure 8:**
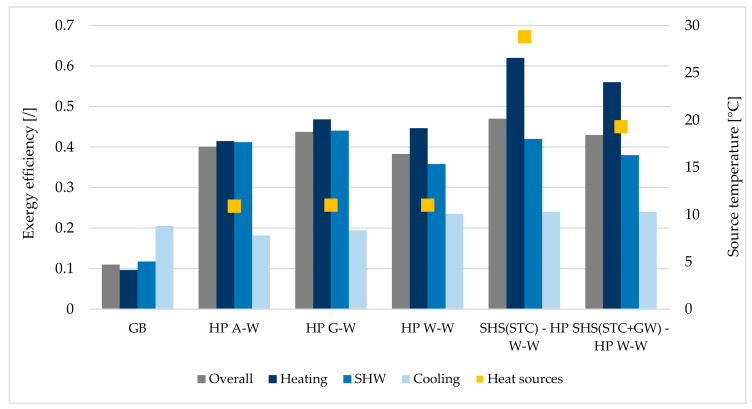
Annually averaged exergy efficiencies for space heating and cooling and SHW production for all analyzed systems in relation to annual average temperatures of heat sources (excluding GB system). Overall exergy efficiency includes all three processes.

Annually averaged exergy efficiencies for separate space heating and cooling and SHW production as well as overall exergy efficiencies for all analyzed systems are shown in Figure 8. The best system in terms of overall exergy efficiency (0.47) is the system based on HP W–W, assisted with solar thermal collectors (SHS(STC)–HP W–W). In comparison with SHS(STC + GW)–HP W–W, the SHS without a GW system has a significantly higher average water temperature of water inside SHS, which goes into HP (28.8 °C vs. 19.3 °C). Due to this fact, the partial exergy efficiencies for space heating and SHW production are higher (0.62 vs. 0.56 and 0.42 vs. 0.38). In the case of space cooling, the values are comparable (0.24 vs. 0.24), since the temperature difference of the supply water in HP is not significantly different (Figure 6, annual variation of the supply water temperature to HP for space heating and SHW production for both systems is between 2880 h and 5830 h). 

If one compares systems where only HP is the source of heating or cooling energy, the overall exergy efficiency is not comparable (HP AW: 0.40, HP GW: 0.44 and HP WW: 0.38), despite similar average annual temperatures of ambient air (10.9 °C), ground (11.0 °C) and ground water (11.0 °C). Based on Figure 5, the lowest source temperatures (air and ground) occur during the heating season, while ground water temperatures change least during the year and are actually higher than the ground temperature during the heating season. The latter explains the maximum exergy efficiency for cooling, because in the case of HP W–W (0.24) it is significantly higher than the other two (HP G–W 0.19 and HP A–W 0.18). It is expected that the system based on natural gas boiler (GB) will have the lowest exergy efficiencies (overall, space heating, SHW production), which are significantly lower compared to efficiencies of other systems. In the case of space cooling, an auxiliary air-to-air vapor-compression cooling system, with ambient air as the heat sink, has been considered. The exergy efficiency of this type of unit is comparable to all other HP-based systems (0.21 vs. 0.18—0.24 for HP-based systems).

### 4.3. Exergy Efficiency—Monthly Averaged

**Figure 9:**
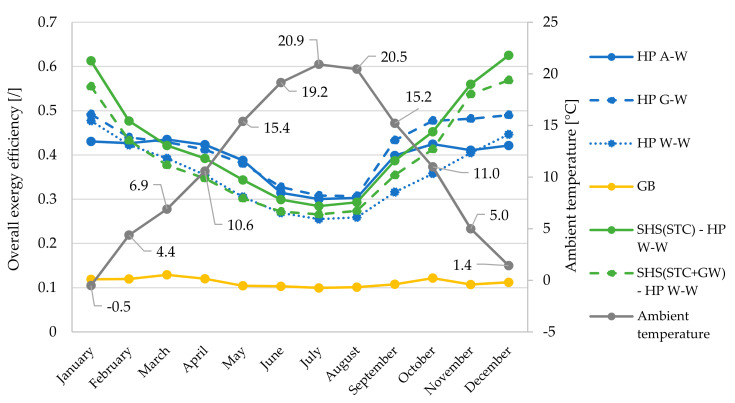
Monthly averaged overall exergy efficiencies of all analyzed systems in relation to monthly averaged ambient air temperature (dead state).

**Figure 10:**
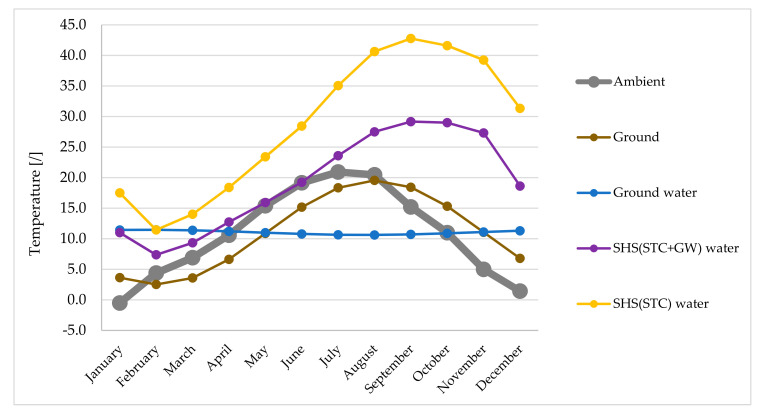
Monthly averaged heat source temperatures: ambient air, ground, ground water and water inside SHS(STC+GW) and SHS(STC).

Monthly averaged overall exergy efficiencies (Figure 9) for space heating and SHW production are presented in relation to monthly averaged heat source temperatures (Figure 10).

In terms of overall exergy efficiency, the greatest differences between the systems are seen in the winter period (November, December, January). During this period, the system based on HP W–W with SHS(STC) proves to be the most efficient system. The difference in efficiency values compared to the HP A–W system is up to 21 percentage points (0.63 vs. 0.42). The reason for this difference is shown in Figure 10, as the largest differences between source temperatures occur in late autumn or early winter. In the months of March–September, the most efficient systems are based on HP A–W and G–W, as both systems are supported by a small temperature difference of the heat sources or sinks and all other HP systems also have pumps, which reduces the exergy efficiency. This is also one of the main reasons for the minimum values of the overall exergy efficiencies of the HP W–W, as we took into account the pump with a power of 350 W when calculating the efficiency.

In September, the temperature differences between the heat sources start to increase significantly, so that the HP W–W based system with SHS(STC + GW) has better overall exergy efficiencies (most noticeable in November and December). 

Figure 10 shows that the largest temperature difference of all heat sources is significantly larger in autumn compared to winter (29.9 vs. 18.0 K), since in summer heat is transferred to the SHS but there is no demand for heat (does not apply to SHW production). The smallest differences between the source temperatures occur in March and April (10.4 and 11.8 K, respectively) and increase later to the maximum difference of 32.1 K in September.

**Figure 11:**
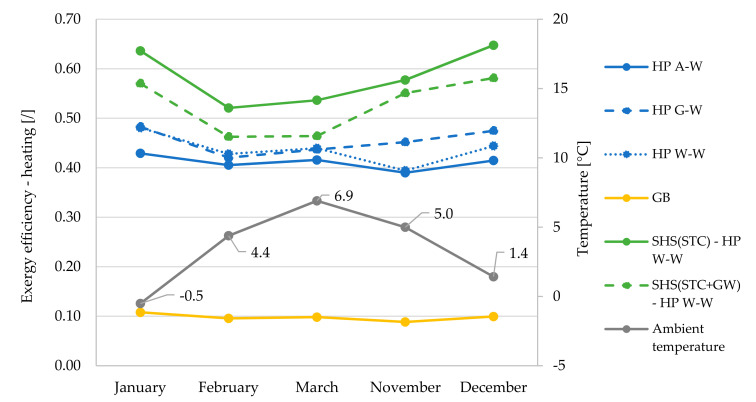
Monthly averaged exergy efficiencies for space heating of all analyzed systems in relation to monthly averaged ambient air temperature (dead state).

**Figure 12:**
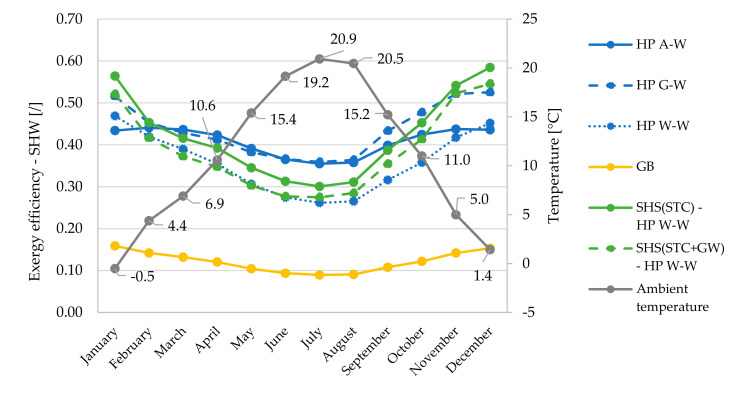
Monthly averaged exergy efficiencies for SHW production of all analyzed systems in relation to monthly averaged ambient air temperature (dead state).

Looking at the exergy efficiency of the space heating process (Figure 11), it can be seen that systems with SHS(STC) and SHS(STC + GW) have significantly higher efficiencies compared to systems with stand-alone HPs. The lower overall efficiency, especially in the case of the system with SHS(STC + GW), is mainly due to the lower exergy efficiency during SHW production (Figure 12). Systems with HP AW and GW have to raise the temperature of the heat to a much higher level than the system with SHS. Again, there is a trend of reducing the differences in efficiencies between all HP systems, which occurs when the ambient temperature increases (dead state).

In the production of SHW (Figure 12), we can observe a greater influence of the numerator describing exergy of heat compared to a higher electricity consumption–exergy, due to lower COP. SHW is produced at a temperature of 55 °C. The greater the difference between the SHW production temperature and the source temperature, the greater the proportion of exergy that is converted to heat, despite the increasing use of pure exergy (electricity) to drive the HP compressor.

## 5. Conclusions

This paper presents an exergy-efficiency analysis of four different systems for the production of heating and cooling energy in a single-house dwelling. The first system is based on a NG boiler for heating purposes, while the cooling energy is provided by an additional air–air vapor-compression cooling device. All three remaining systems are based on HPs, while in the case of the third and fourth systems, a SHS tank is connected to a W–W HP. The main objective of this study was to compare the overall exergy efficiencies of all four systems (including space heating and cooling and SHW production). The final conclusions of the above-mentioned exergy analyses are as follows:

- The system based on the NG boiler for space heating and SHW production purposes has up to 36% percentage points lower overall annual exergy efficiency compared to the most efficient system (0.11 vs. 0.47—SHS (STC)–HP W–W). This shows an enormous waste of exergy when NG is burned for heating purposes only. From the point of view of overall energy (and exergy) efficiency, it would be much more sensible to promote systems such as combined heat and power plant (electrical energy + heat production). The other possible systems that are far more energy efficient are based on natural gas-driven heat pumps and hybrid heat pumps. In the latter case, natural gas can be used as a heat source, while, in the former case, burning natural gas represents chemical energy that can be converted into mechanical work in the compressor.
- The system based on SHS in combination with STC (SHS (STC)–HP W–W) was identified as one of the most efficient systems in terms of overall exergy efficiency. The main factor contributing to higher efficiency values is higher water temperatures within SHS, as this is the heat source for the HP W–W. Compared to ambient air, ground and ground water, the annual average water temperature within the SHS was significantly higher (10.9 vs. 11.0 vs. 11.0 vs. 28.8 K).
- Special attention should be paid to auxiliary pumps in real systems, as electrical energy is pure exergy and represents direct exergy losses (entropy generation). For example, HP A–W does not require an auxiliary water pump, unlike HP W–W and HP G–W (with a rated electrical power of 350 W and 50 W respectively) and systems with SHS(STC) and SHS(STC + GW)-rated electrical outputs of 50 W and 50 + 50 W, respectively. Therefore, the STC and GW based systems have achieved an overall energy efficiency of 0.43, which is directly comparable to the efficiency of the system with a HP G–W (0.44) and is close to the system based on HP A–W (0.40). However, the system based on SHS(STC+GW) represents an enormous energy saving by using the waste heat from grey water, as described in more detail in the Addendum.

## 6. Addendum

The primary aim of this study was not to consider the analysis of CO_2_ emissions of the presented systems for the production of heat or cooling energy. Therefore, within the Addendum a comparison of all the systems that are suitable for the production of heating and cooling energy in terms of annual CO_2_ emissions is presented. Within this analysis, the annual energy use of household sector for heating and cooling purposes and known emission factors (EF) for Slovenia (EF for natural gas (for year 2015) and electricity (for year 2018) emission factors are 0.2 kgCO_2_/MWh and 0.353 kgCO_2_/MWh, respectively) have been considered. Compared to a system based on combined the NG boiler and auxiliary cooling device, the system with SHS(STC) provides a reduction in CO_2_ emissions of up to 64%, while the system based on HP W–W can reduce emissions by up to 54%. 

In terms of actual numbers, the annual emissions of a system based on the NG boiler are 1.59 tonnes of CO_2_, while the emissions in the case of systems with HP A–W, W–W, G–W, SHS (STC + GW) and SHS (STC) are 0.70, 0.73, 0.64, 0.65 and 0.57 tonnes of CO_2_ regarding the case building presented in this article. If we take those numbers into the context of the overall Slovenian emission of CO_2_ in 2017 with 15.59 million tonnes of CO_2_, the total CO_2_ emissions of 824,618 households (year 2018) based on NG (with auxiliary cooling device) would be 2.22 million tonnes of CO_2_. The most efficient system—with HP W–W and SHS(STC)—could reduce this number by up to 1.42 million tonnes of CO_2_ annually. Considering the overall Slovenian emissions in all sectors, the reduction in CO_2_ at a national level, if using a system based on SHS(STC), could be up to 9.1%.

## Figures and Tables

**Table 1:** Construction properties for standard and low-energy building.

Dimension	Value
U—outside wall	0.142 W/m^2^K
U—roof	0.155 W/m^2^K
U—inner wall	0.426 W/m^2^K
U—floor to ground	0.211 W/m^2^K
U—glazing	1.4 W/m^2^K
g of glazing	0.62
Infiltration as air exchange per hour	0.2 h^−1^
Heat gains	5 W/m^2^
Heating set point	20 °C
Cooling set point	25 °C

**Table 2:** Characteristic of heating and cooling energy generators.

Type of Heat/Cold Generator	Characteristic of Generator
Air—water heat pump [23]	${\dot{Q}}_{h}\;=\;7.6\;\mathrm{kW}$, COP = 3.94 at A2/W35
	${\dot{Q}}_{c}\;=\;6.2\;\mathrm{kW}$, EER = 2.76 at A35/W7
Ground (brine)—water heat pump [23]	${\dot{Q}}_{h}\;=\;5.3\;\mathrm{kW}$, COP = 4.10 at B0/W35
	${\dot{Q}}_{c}\;=\;6.2\;\mathrm{kW}$, EER = 5.55 at B10/W7
Water—water heat pump [23]	${\dot{Q}}_{h}\;=\;7.3\;\mathrm{kW}$, COP = 5.37 at W10/W35
	${\dot{Q}}_{c}\;=\;6.3$, EER = 5.68 at W10/W7
Natural gas boiler [24]	$\eta_{\mathrm{gas},a}=87.5\%$
Auxiliary air—air vapor-compression cooling system [22]	EER = 2.5 at A35/W7

**Table 3:** Characteristics of solar thermal collectors [26].

Characteristic	Value
Type	Flat plat collector
Length × Width (mm)	2170 × 1170
Intercept efficiency	0.79
Efficiency slope (W/m^2^K)	4.03
Efficiency curvature (W/m^2^K^2^)	0.0107

**Table 4:** Main components employed in the TRNSYS simulation.

Component	TRNSYS Type
Building	TYPE 56
Solar collector	TYPE 1
Seasonal and SHW storage tank	TYPE 4
Weather and data reader	TYPE 9
Pump, fan	TYPE 3
Heat pump—modelled by multi-dimensional data interpolation	TYPE 581
Natural gas boiler	TYPE 700
ON/OFF Differential controller	TYPE 2
Solar radiation processor	TYPE 16
Soil temperature profile	TYPE77
Heat exchanger, recuperator	TYPE 91
Daily, annual occupation	TYPE 14
Ventilation system control	TYPE 40
Equation editor	EQUA

## Data Availability

No new data were created or analyzed in this study. Data sharing is not applicable to this article.

## Abbreviations

A–W	Air—water heat pump
ACH	Air-change rate
COP	Coefficient of performance
G–W	Ground—water heat pump
GW	Grey water unit
HP	Heat pump
NG	Natural gas
PV/T	Photovoltaic/thermal
RES	Renewable energy sources
SCW	Sanitary cold water
SHS	Seasonal heat storage
SHW	Sanitary hot water
SPF	Seasonal performance factor
STC	Solar thermal collectors
W–W	Water—water heat pump

## Nomenclature

e	Specific exergy (J/kg)
E	Exergy (J)
H	Calorific value (J/kg)
Q	Heat (J)
T	Temperature (K)
Greek Letters	
η	Efficiency (/)
Subscripts	
a	Ambient
c	Cooling
CH_4_	Methane
ex	Exergy
h	Heating
in	Input
ov	Overall
s	Superior
shw	Sanitary hot-water
